# Coextrusion-Based 3D Plotting of Ceramic Pastes for Porous Calcium Phosphate Scaffolds Comprised of Hollow Filaments

**DOI:** 10.3390/ma11060911

**Published:** 2018-05-29

**Authors:** In-Hwan Jo, Young-Hag Koh, Hyoun-Ee Kim

**Affiliations:** 1 School of Biomedical Engineering, Korea University, Seoul 02841, Korea; jojotan@naver.com; 2 Department of Materials Science and Engineering, Seoul National University, Seoul 08826, Korea; kmhe@snu.ac.kr

**Keywords:** calcium phosphate, additive manufacturing, porous structure, mechanical properties, biocompatibility

## Abstract

This paper demonstrates the utility of coextrusion-based 3D plotting of ceramic pastes (CoEx-3DP) as a new type of additive manufacturing (AM) technique, which can produce porous calcium phosphate (CaP) ceramic scaffolds comprised of hollow CaP filaments. In this technique, green filaments with a controlled core/shell structure can be produced by coextruding an initial feedrod, comprised of the carbon black (CB) core and CaP shell, through a fine nozzle in an acetone bath and then deposited in a controlled manner according to predetermined paths. In addition, channels in CaP filaments can be created through the removal of the CB cores during heat-treatment. Produced CaP scaffolds had two different types of pores with well-defined geometries: three-dimensionally interconnected pores (~360 × 230 μm^2^ in sizes) and channels (>100 μm in diameter) in hollow CaP filaments. The porous scaffolds showed high compressive strengths of ~12.3 ± 2.2 MPa at a high porosity of ~73 vol % when compressed parallel to the direction of the hollow CaP filaments. In addition, the mechanical properties of porous CaP scaffolds could be tailored by adjusting their porosity, for example, compressive strengths of 4.8 ± 1.1 MPa at a porosity of ~82 vol %. The porous CaP scaffold showed good biocompatibility, which was assessed by in vitro cell tests, where several the cells adhered to and spread actively with the outer and inner surfaces of the hollow CaP filaments.

## 1. Introduction

Calcium phosphate (CaP) ceramics are one of the most promising biomaterials for reconstruction of bone defects in dental, maxillofacial, and orthopedic applications, since they can have excellent biocompatibility and bioactivity owing to their chemical compositions similar to inorganic phase of natural bones [1,2]. These materials in the form of paste have been extensively used as the bone cements in orthopedic surgery, which can be molded or injected into bone defects and implants sites, followed by in situ setting and hardening [3]. In addition, antibacterial agents (e.g., silver) can be incorporated into CaP pastes, which can be effective for the prevention and treatment of infections [4]. In particular, when formulated into porous structure, porous CaP scaffolds can provide a favorable environment for cellular reaction and colonization by osteoblasts, thus stimulating bone ingrowth into pores and bone regeneration in osseous sites [5]. Fundamentally, the bone regeneration ability and mechanical functions of CaP scaffolds strongly depend on their porous structure, such as porosity, pore size, and pore interconnectivity, as well as pore configuration [6,7].

In recent years, additive manufacturing (AM) techniques have gained increasing interest for the production of porous ceramic and metallic scaffolds, since they can construct 3D anatomical external shapes with internal porous structures tailored for individual patients using computer-aided design (CAD) [8,9,10,11,12,13,14]. This great ability, the mechanical properties of porous ceramic scaffolds, can be significantly enhanced and tailored for specific applications, while, at the same time, inducing excellent bone regeneration when used as bone scaffolds [15,16,17,18,19]. There are a variety of ceramic-based AM techniques, which can create successive layers of ceramic-based feedstocks using their own consolidation mechanisms. These include the 3D deposition of ceramic green filaments (e.g., direct-ink-write assembly [15,20,21], robocasting [19,22,23], extrusion freeforming [24,25,26], freeze-form extrusion fabrication [16,27], rapid direct deposition of ceramic paste [28], and ceramic/camphene-based 3D extrusion [29,30]), photo-curing of ceramic slurries (e.g., stereolithography (SLA) [31] and digital light processing (DLP) [32]), and polymer jetting onto powder layers [33,34]. For example, extrusion-based AM techniques can construct 3D pore networks by continuously depositing green ceramic filaments extruded through fine nozzles according to predetermined paths [10]. Thus, the porosity and pore size of porous ceramic scaffolds can be readily tuned by adjusting the distance between deposited filaments, which can provide mechanical properties tailored for specific applications. In addition, when specially designed nozzles (e.g., coaxial nozzle) are used, porous ceramic scaffolds comprised of hollow filaments can be produced [22,35]. These unique porous structures would be expected to provide high specific strengths and large surface area compared to those comprised of dense filaments [30]. However, the ability to control the cross-sectional geometry of hollow filaments should be improved to enhance the structural stability and mechanical properties of porous scaffolds.

In this study, we proposed coextrusion-based 3D plotting of ceramic pastes (CoEx-3DP) as a new type of additive manufacturing (AM) technique, which can produce porous calcium phosphate (CaP) ceramic scaffolds comprised of hollow CaP filaments with controlled channel geometry and size. This technique can combine the principles of coextrusion process for the creation of green ceramic filaments with a core/shell structure [36,37] and extrusion-based AM technique using a ceramic paste for the rapid deposition of extruded filaments [28]. In particular, carbon black (CB) is used as the fugitive material to create channels in hollow CaP filaments after heat-treatment. This innovative approach allowed for the construction of periodic hollow filaments with a controlled cross-sectional geometry using a commercial single nozzle. The porous structures of produced CaP scaffolds and microstructure of hollow CaP filaments were evaluated. Mechanical properties were characterized by compressive strength tests. The in vitro biocompatibility was also examined using a pre-osteoblast cell line to evaluate the potential of porous CaP ceramics as the bone scaffold for the replacement of cancellous bones. The processability and utility of the present method for the production of porous CaP scaffolds are also discussed in this paper.

## 2. Materials and Methods

### 2.1. Principle of CoEx-3DP Technique

**Figure 1:**
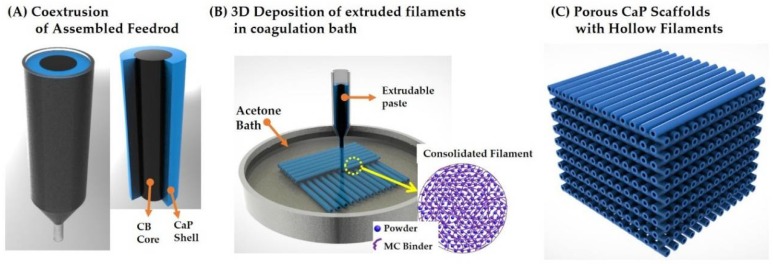
Schematic diagrams showing CoEx-3DP technique for the production of porous CaP scaffolds comprised of hollow filaments: (**A**) the coextrusion of an assembled feedrod with the CB core and CaP shell; (**B**) 3D deposition of extruded filaments in a congratulation bath; and (**C**) porous CaP scaffolds comprised of hollow filaments.

Figure 1 shows the schematic diagram of CoEx-3DP technique for the production of porous CaP scaffolds comprised of hollow filaments. For this goal, green filaments with a tightly controlled cross-sectional profile were produced by coextruding a feedrod comprised of a core (carbon black (CB) paste) and a shell (CaP paste) through a single nozzle (Figure 1A). Then, an assembled feedrod was extruded through a single nozzle in a coagulation bath and then deposited according to predetermined paths at a stacking sequence of 0°/90° (Figure 1B). The produced green scaffolds were heat-treated to remove the binder and CB core, followed by sintering at 1250 °C to density hollow CaP filaments (Figure 1C). 

### 2.2. Starting Materials

As the CaP ceramic, commercially available biphasic calcium phosphate (BCP; OssGen Co., Daegu, Korea), comprised of hydroxyapatite (HA) and β-tricalcium phosphate (β-TCP) with a weight ratio of 60:40, was used owing to their excellent biocompatibility and bioactivity in vitro and in vivo [1,2]. The particle sizes of the BCP powders were ~0.5–0.8 μm. Commercial carbon black (CB) powder (Cabot Black Pearls BP-120; Cabot Corp., Boston, MA, USA) was used as the fugitive material. Methylcellulous polymer (MC; Sigma Aldrich, St. Louis, MO, USA) and an oligomeric polyester dispersant (Hypermer KD-6; UniQema, Everburg, Belgium) were used as the binder and dispersant, respectively, for the preparation of CaP and CB pastes.

### 2.3. CaP and CB Pastes Preparation

To prepare CaP and CB pastes, an aqueous MC solution with a concentration of 5 wt % was first prepared by dissolving MC polymer in water by magnetic stirring. Afterwards, predetermined amounts of CaP or CB powders were added to the MC solutions and vigorously mixed using a paste mixer (Hantech Co, Ltd., Gyeonggi-do, Korea). A small amount of the dispersant was also added to improve the dispersion of the powders in the pastes. The compositions of CaP and CB pastes used in this study are summarized in Table 1. The rheological behaviors of the CaP and CB pastes were characterized using a cone and plate type rheometer (Discovery HR-2, TA instrument, Newcastle, DE, USA).

### 2.4. Initial Feedrod Preparation

For the CoEx-3DP process, an initial feedrod was prepared by assembling a CB core and CaP shell (cf. Figure 1A). A CB core was prepared by casting the CB paste into a mold with a diameter of 6.5 mm at room temperature and then placed at 60 °C for gelation. Then, the CB core was removed from the mold and placed at the center of a mold with diameter of ~15 mm. The CaP paste was then cast into the mold with the CB core at room temperature and then kept at 37 °C to stabilize the assembled feedrod for the CoEx-3DP process.

### 2.5. Porous Green Scaffolds Production

**Figure 2:**
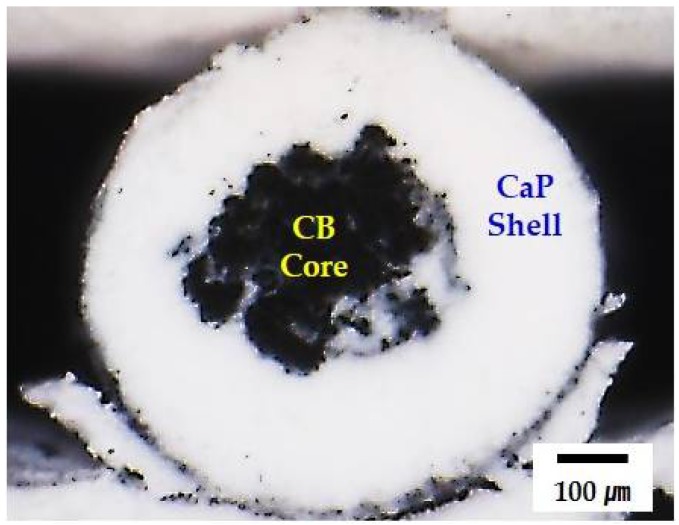
Representative optical image of the extruded filament in acetone, showing its core/shell structure.

Porous green scaffolds comprised of filaments with the CB core and BCP shell structure were produced by continuously depositing the initial feedrod according to predetermine paths at a stacking sequence of 0°/90° (cf. Figure 2B). More specifically, the assembled feedrod was extruded through a fine nozzle with a diameter of ~500 μm under a pressure of ~150 kPa. Then, the extruded filaments were deposited at a constant deposition speed of 10 mm/s in an acetone bath at room temperature using a computer-controlled robot (Ez-ROBO5, Iwashita, Japan). The produced green scaffolds were dried at 60 °C for 24 h to remove water. Thereafter, the porous scaffolds were directly heated up to 1250 °C at a heating rate of 5 °C /min, in which the MC polymer and CB were removed. These were maintained at this temperature for 2 h to densify the CaP filaments. To control the porosity and mechanical properties of porous CaP scaffolds comprised of hollow filaments, the distance between deposited green filaments was adjusted (i.e., 0.35 mm, 0.5 mm, and 0.75 mm).

### 2.6. Porous Structure, Microstructure, and Crystalline Structure Characterization

The porous structures of porous CaP scaffolds and microstructure of hollow CaP filaments were characterized by field emission scanning electron microscopy (FE-SEM; JSM-6701F; JEOL Techniques, Tokyo, Japan). The overall porosity (*p*) of the porous CaP scaffold was calculated by considering its apparent density (*ρ*_a_) divided by the theoretical density (*ρ*_s_) of the dense CaP ceramic, as follows:

*P* (%) = 100 − 100·(*ρ*_a_/*ρ*_s_) (1)

The apparent density of the scaffold was computed by measuring the mass (m) and volume (V) (i.e., *ρ*_a_ = m/V). The theoretical density of the dense CaP ceramic was taken to be 3.14 g/cm^3^ [1]. The fractions of the 3D pores and channels were roughly calculated by considering the FE-SEM images of the samples. The fractions of the 3D pores and channels were roughly calculated by considering their area appearing in the FE-SEM images of the samples. Five samples were examined for this measurement and three images were taken from each sample. The crystalline structures and phases of the porous CaP scaffolds were characterized by X-ray diffraction (XRD, M18XHF-SRA, MacScience Co., Yokohama, Japan).

### 2.7. Compressive Strength Tests

Compressive strength tests were used to evaluate the mechanical properties of the porous CaP scaffolds comprised of hollow filaments. Specimens with dimensions of ~11 × 11 × 11 mm^3^ were unidirectionally compressed at a crosshead speed of 0.5 mm/min using a screw driven load frame (OTU-05D; Oriental TM Corp., Siheung, Korea). Compression load was applied parallel or normal to the direction of hollow CaP filaments. During the tests, the stress and strain responses of the specimens were monitored. The compressive modulus was computed from the slop of the initial linear portion of the stress–stain curve. Eight specimens were tested to obtain the mean value and standard deviation.

### 2.8. In Vitro Biocompatibility Tests

The in vitro cytocompatibility of the porous CaP scaffolds comprised of hollow filaments was evaluated using a pre-osteoblast cell line (MC3T3-E1; ATCC, CRL-2593, Rockville, MD, USA) [38]. For comparison purpose, tissue culture plate (Falcon™ Standard Tissue Culture Dishes, USA) was used as the positive control. Prior to the cell seeding, the samples were sterilized with 70% ethanol for 6 h and washed with phosphate buffered saline (PBS). Afterwards, the samples were dried on a clean bench under ultraviolet (UV) irradiation for 24 h.

The preincubated cells were plated at a density of 5 × 10^4^, 3 × 10^4^, and 3 × 10^4^ cells/mL for the initial cell attachment, proliferation and differentiation tests, respectively. A 12-well culture plate (SPL Life Sciences Co., Ltd., Gyeonggi-do, Korea) with walls having a diameter of 20 mm and a depth of 18 mm was used for this evaluation. The MC3T3-E1 cells were cultured in a humidified incubator in an atmosphere containing 5% CO_2_ at 37 °C. A minimum essential medium (α-MEM: Welgene Co., Ltd., Seoul, Korea) supplemented with 10% fetal bovine serum (FBS) and 1% penicillin–streptomycin, as well as 10 mM β-glycerophosphate (Sigma) and 10 µg mL^−1^ ascorbic acid was used as the culturing medium [38]. After one day of cell seeding, the osteogenic differentiation culture media was used for ALP activity measurements. In addition, the culture medium was changed every three days.

The morphologies of the attached cells on the porous CaP scaffolds comprised of hollow filaments after 6 h of culture were examined by confocal laser scanning microscopy (CLSM; C1 PLUS, Nikon, Tokyo, Japan). For CLSM observations, the cells were fixed with 4% paraformaldehyde, permeabilized with 0.1% Trion X-100, and stabilized with 1% BSA. The actin filament and nuclei of the cells were dyed with 2.5% Alexa Fluor 546 phalloidin (Molecular Probes, Eugene, OR, USA) and ProLong Gold antifade reagent with DAPI (Molecular Probes, Eugene, OR, USA), respectively. The stained substrates were placed on a cover slide without further treatments, and the cell morphology was observed in air.

The cell proliferation rate was examined using a MTS (methoxyphenyl tetrazolium salt) assay with 3-(4,5-dimethylthiazol-2-yl)-5-(3-carboxymethoxyphenyl)-2-(4-sulfophenyl)-2H-tetrazolium (MTS, Promega, Madison, USA) for mitochondrial reduction. The quantity of the formazan product was measured by the absorbance at 490 nm using a micro-reader (Model 550; Biorad, Hercules, CA, USA). Five samples were tested for each test.

The degree of cell differentiation was assessed using an alkaline phosphatase (ALP) activity test, in which 10 mM β-glycerophosphate (β-GP) and 50 µg/mL ascorbic acid (AA) were added to the culture medium. After culturing for 1, 3, and 6 days, p-nitrophenyl phosphate (pNPP) production was colorimetrically measured at an absorbance of 405 nm using a micro reader (Model 550; Biorad, USA). Five samples were tested for each test.

### 2.9. Polymer-Infiltrated Scaffold Production

To demonstrate the utility of the porous CaP scaffolds comprised of hollow filaments, the channels in the hollow CaP filaments were filled with poly(ε-caprolactone) (PCL) polymer. PCL has been widely used as bone scaffolds owing to its good biocompatibility, biodegradability, and excellent mechanical properties [39]. To achieve this goal, the porous scaffold was first immersed in liquid paraffin wax (Sigma Aldrich, St. Louis, MO, USA) at 85 °C and cooled down to room temperature. This allowed the 3-dimensionally interconnected pores (cf. Figure 1) to be filled with the wax, while the channels in the hollow filaments could be preserved. Afterwards, the outer surfaces of the samples were gently ground to expose the channels in the hollow filaments. The sample was then immersed in caprolactone oligomer (UVIAK RC-223, Aekyung Chemical Co., Ltd., Korea) containing 1.5 wt % of benzoyl peroxide (Sigma Aldrich, St. Louis, MO, USA) as the thermal initiator, followed by heat-treatment 85 °C for thermal curing and removal of the liquid wax. This approach allowed the channels in the hollow filaments to be filled with biocompatible PCL polymer, while preserving its 3D pore networks.

### 2.10. Statistical Analysis

All experimental results are expressed as the mean ± standard deviation (SD). The difference between the two groups was determined using a one-way analysis of variance (ANOVA). *p* < 0.05 and *p* < 0.01 were considered to be statistically significant (* *p* < 0.05 and ** *p* < 0.01).

## 3. Results and Discussion

### 3.1. Designing of Feedrod with Core/Shell Structure for Coextrusion 

To make full use of CoEx-3DP process, which was newly developed in this study, special care should be taken to design an initial feedrod with a core/shell structure to be coextruded simultaneously in a controlled manner. More specifically, the CB core and CaP shell should have similar extrusion behaviors during coextrusion through a single nozzle, and thus green filaments with a controlled, uniform core/shell structure can be produced. In particular, a relatively high CaP content of 30 vol % was used for the preparation of a CaP paste to achieve high densification after sintering at high temperatures [28]. However, in the case of CB pastes, the use of relatively high CB contents (e.g., >20 vol %) would not provide favorable extrusion through a fine nozzle. Thus, four different types of CB pastes with various CB contents (8, 10, 12, and 15 vol %) were examined as the core material. It was observed that the core/shell thickness ratio increased remarkably with a decrease in CB content, presumably due to a decrease in the viscosity of the CaP paste (data not shown here). When a CB content of 10 vol % was used, the hollow filament showed a core/shell thickness ratio similar to that (0.5) of the original design. Thus, this composition was used for the production of porous CaP scaffolds (cf. Table 1). This finding suggests that the use of the initial feedrod, comprised of the CB core and CaP shell with extrusion behaviors tailored for coextrusion, allows for the creation of green filaments with a tightly controlled core/shell structure.

### 3.2. Geometry and Microstructure of Green Filaments

The geometry and microstructure of the solidified filaments with the CB core and CaP shell structure were examined by optical microscopy and SEM. A round morphology was well preserved owing to the rapid solidification of the extruded filament in acetone through solvent extraction (Figure 2). In addition, no noticeable defects, such as interfacial delamination or cracks associated with drying shrinkage, were observed. The interface between the CB core and BCP shell is indicated by the dashed line. This finding suggests that both BCP and CB pastes used as the shell and core, respectively, can be solidified simultaneously, thus allowing for the creation of green filaments with a controlled core/shell structure.

**Figure 3:**
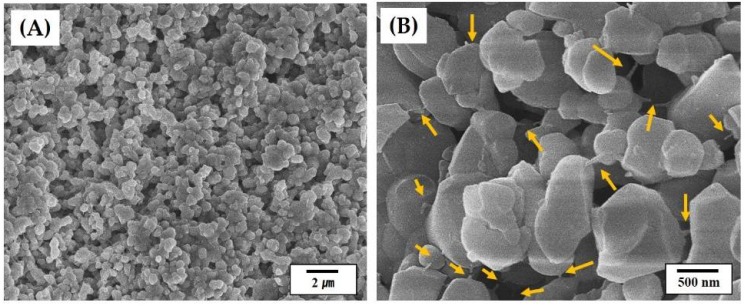
Representative SEM images of the CaP shell at: (**A**) low magnification; and (**B**) high magnification. Arrows in (**B**) indicate the MC polymer used as the binder.

Figure 3A,B shows representative SEM images of the CaP shell at low and high magnifications, respectively. The CaP powders were uniformly distributed throughout the filament (Figure 3A). No large voids were observed. In addition, the MC polymer, indicated by arrows, could effectively bind the CaP powders, thus providing good strength for structural stability (Figure 3B). This finding suggests that the CaP powders with a high content of 30 vol % can be uniformly mixed with an aqueous MC solution and preserve uniform distribution during the precipitation of the MC polymer through solvent extraction in acetone [28,40,41]. This would allow for the achievement of the high densification of the CaP shells after sintering at high temperatures.

### 3.3. 3D Porous Structure of Green Scaffolds

**Figure 4:**
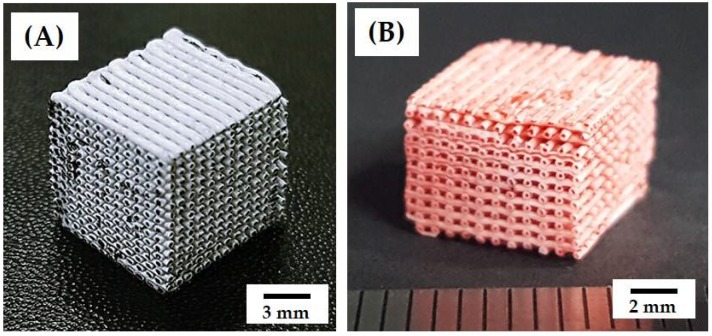
Representative optical image of: (**A**) the green scaffold after drying at 60 °C to remove water; and (**B**) the porous CaP scaffold comprised of hollow filaments after heat-treatment at 1250 °C for 2 h. For visualization, the porous CaP scaffold was dyed red.

Porous green scaffolds, comprised of filaments with the CB core and CaP shell, were successfully produced by continuously depositing extruded filaments with a core/shell structure in an acetone bath according to predetermined designs. Figure 4A shows representative optical image of the green scaffold after drying at 60 °C to remove water. For visualization, the outmost surfaces of the porous scaffold were slightly ground to reveal the CB cores. The produced scaffold showed good shape tolerance without noticeable defects, such as the collapsing or cracking of the green filaments. A linear shrinkage of ~14.2% was observed after drying at 60 °C. This finding suggests that the use of the assembled feedrod, comprised of the CB core and BCP shell, for coextrusion and solvent extraction mechanism for the rapid consolidation of the extruded filaments enables the production of porous scaffolds, comprised of filaments with a tightly controlled core/shell structure.

### 3.4. 3D Porous Structure of Porous Scaffolds with Hollow Filaments

Porous CaP scaffolds, comprised of hollow CaP filaments, were produced after heat-treatment at 1250 °C for the removal of the CB cores and densification of the CaP shells. The porous scaffold well preserved the three-dimensionally interconnected pore networks without noticeable defects, such as distortion or cracking, as shown in Figure 4B. It should be noted that the CB cores in the green filaments could be readily and completely removed without destroying thin CaP shells during heat-treatment, which is one of the most striking advantages of CB as the fugitive material [36,37]. A linear shrinkage of ~31.7% was observed after sintering at 1250 °C for 2 h. The dimensions of the green scaffold and porous CaP scaffold after sintering at 1250 °C for 2 h are summarized in Table 2.

**Figure 5:**
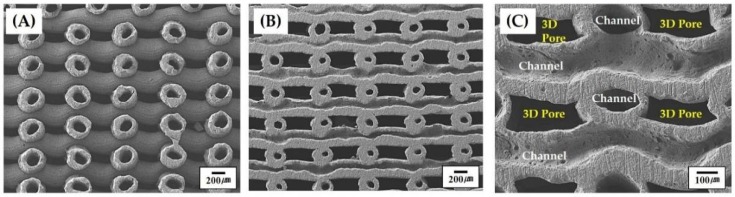
Representative SEM images of the porous CaP scaffold comprised of hollow filaments: (**A**) the porous structure of the as-produced scaffold; and the construction of the three dimensionally interconnected pores and channels in the hollow filaments at (**B**) low and (**C**) high magnifications. For visualization (Figure 5B,C), the outmost surfaces of the porous scaffold were slightly ground to reveal the channels.

The porous structures of the porous CaP scaffolds comprised of hollow filaments were more closely examined by SEM, as shown in Figure 5A–C. Hollow CaP filaments with a uniform cross-sectional geometry were constructed in a periodic pattern throughout the porous scaffold (Figure 5A). It should be noted that the porous scaffold can have three-dimensionally interconnected pores, which would provide a favorable environment for bone ingrowth when used as the bone scaffold. The construction of channels in the CaP filaments is more closely visible in Figure 5B. For visualization, the outmost surfaces of the porous scaffold were slightly ground to reveal the channels. The straight channels were constructed at an orientation of 0°/90°. In addition, the hollow CaP filaments were well bonded together without noticeable interfaces (Figure 5C), which is one of the most striking advantages of the newly developed CoEx-3DP technique. 

It should be noted that the three-dimensionally interconnected pores—360 × 230 μm^2^ in size (Table 3)—would be expected to provide a favorable 3D spaces for bone ingrowth when used as bone scaffolds [6,7,40,41]. The channels with sizes larger than 100 μm would be also beneficial to bone regeneration.

### 3.5. Overall Porosity and Fraction of 3D Pores

The porous scaffold showed a relatively high porosity of 73 ± 1.5 vol %, which was calculated by considering its apparent density (*ρ*_a_) divided by the theoretical density (*ρ*_s_) of the dense CaP ceramic. The fraction of the 3D pores, roughly computed from the SEM image of the scaffold, was ~62 vol %. It should be noted that the fraction of 3D pores can be readily tuned by adjusting the distance between hollow filaments. Thus, the overall porosity of BCP scaffolds can be tailored for specific applications. 

### 3.6. Microstructure of Hollow CaP Filaments

**Figure 6:**
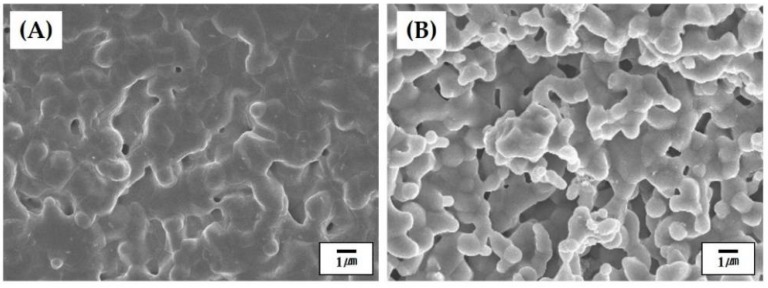
Representative SEM images of (**A**) the outer and (**B**) inner surfaces of the hollow CaP filament.

Figure 6A,B shows representative SEM images of the outer and inner surfaces of the hollow CaP filament, respectively. The outer surface of the filament showed a relatively dense microstructure with small residual pores (Figure 6A), suggesting that the CaP shell could be well densified at 1250 °C for 2 h. On the other hand, the inner surface of the hollow filament, which was originally contacted with the CB core, revealed a rough, porous microstructure (Figure 6B). It is reasonable to suppose that the rough surface would be formed due to the partial mixing of the CaP and CB pastes at the interface during coextrusion process. However, it should be noted that the rough surface would be beneficial to cell attachment, proliferation, and differentiation when used as the bone scaffold [6,41].

### 3.7. Crystalline Phases of CaP Scaffold

**Figure 7:**
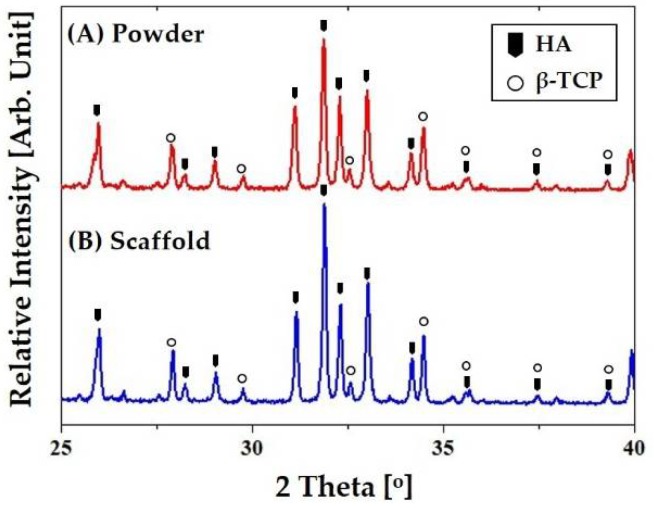
Representative XRD patterns of: (**A**) the CaP powder; and (**B**) porous CaP scaffold comprised of hollow filaments after sintering at 1250 °C for 2 h.

The crystalline structures and phases of the porous CaP scaffolds comprised of hollow filaments were characterized by XRD. For comparison purposes, the CaP powder was also tested. Figure 7A,B shows representative XRD patterns of the CaP powder and porous CaP scaffold, respectively. Both samples showed very similar XRD patterns, where only peaks corresponding to crystalline HA (JCPDS file No. 09-0432) and β-TCP (JCPDS file No. 09-0169) were observed, indicating BCP ceramic. No additional peaks or noticeable changes in the relative intensities of the peaks were observed after sintering at 1250 °C (Figure 7B). This finding suggests that the CB used as the fugitive core can be completely removed during heat-treatment without altering the crystalline structures and phases of the CaP shell.

### 3.8. Mechanical Properties of CaP Scaffold

**Figure 8:**
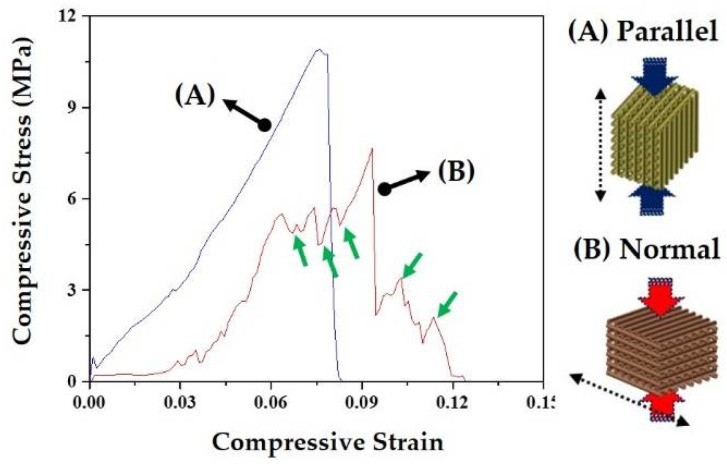
Representative stress versus strain responses of the porous CaP scaffolds comprised of hollow filaments, when compressed (**A**) parallel and (**B**) normal to the direction of hollow firmaments. Arrow in Figure 8B indicate a chaotic regime of decreasing and increasing stress.

The mechanical properties of the porous CaP scaffolds comprised of hollow filaments were characterized by compressive strength tests. The porous scaffolds were compressed parallel or normal to the direction of hollow filaments (insets in Figure 8A,B). Basically, the fracture behavior of the porous scaffold was strongly affected by the alignment of hollow filaments. Figure 8A,B shows representative stress versus strain responses of the porous CaP scaffolds when compressed parallel and normal to the direction of hollow filaments, respectively. When the porous scaffold was compressed parallel to the direction of hollow filaments, the stress increased almost linearly with an elastic response up to ~12 MPa, and then rapidly dropped due to the fracture of the sample, as shown in Figure 8A. On the other hand, when compressed normal to the direction of hollow filaments, the porous scaffold showed a chaotic regime of decreasing and increasing stress, marked by arrows, before reaching peak load (Figure 8B). This would be attributed to the partial fracture of the CaP filaments. For example, the first decrease in stress was due to the fracture of the filament that was broken initially [42,43]. 

It should be noted that the porous CaP scaffold with hollow filaments can have much higher compressive strength when compressed parallel to the direction of hollow filaments, as summarized in Table 4.

**Figure 9:**
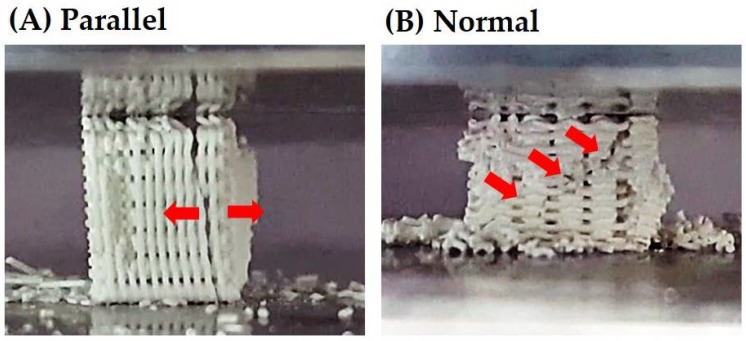
Representative optical images of the porous CaP scaffolds comprised of hollow filaments, when compressed (**A**) parallel and (**B**) normal to the direction of hollow filaments, showing their fracture behavior during the tests.

Figure 9A,B shows representative optical images of the porous BCP scaffold compressed parallel and normal to the direction of hollow filaments, respectively, during compression. When compressed parallel to the direction of hollow filaments, the cracks propagated vertically, thereby splitting the porous scaffold into several parts (Figure 9A). On the other hand, when compressed normal to the direction of hollow filaments, several parties due to the partial fracture of the hollow filaments were detached from the scaffold during the test (Figure 9B). It should be noted that these partial fractures occurred several times before peak load.

The CaP scaffold comprised of hollow filaments has a periodic arrangement of thin CaP shells. Thus, its fracture behavior and mechanical properties should be strongly affected by the loading direction. More specifically, when compressed parallel to the direction of hollow filaments, CaP shells can effectively withstand applied load, thus providing relatively high compressive strengths (cf. Table 4), as is often the case with porous ceramics with periodic pore channels [36,44,45,46]. In addition, the CaP shells can be cracked due to tensile stress developed during compression, thus causing vertical cracking (cf. Figure 9A). On the other hand, when compressed normal to the direction of hollow filaments, CaP shells can be more easily fractured by the bending stress, thus resulting in much lower compressive strengths. However, in this case, some parts due to the fracture of the hollow CaP filaments would be detached from the porous scaffold during compressive strength tests (cf. Figure 9B), thus demonstrating a chaotic regime of decreasing and increasing stress (cf. Figure 8B). It should be noted that the porous CaP scaffold comprised of hollow filaments can have reasonably high compressive strengths of ~12.3 ± 2.2 MPa at the very high porosity of ~73 vol %, thus finding useful applications as the bone scaffold.

One of the most striking advantages of the newly developed CoEx-3DP is the ability to achieve high porosities and high mechanical properties owing the construction of hollow CaP filaments in a periodic pattern. For example, a porous scaffold with a high porosity of ~82 vol % could be produced by increasing the distance between deposited filaments, as summarized in Table 5. 

**Figure 10:**
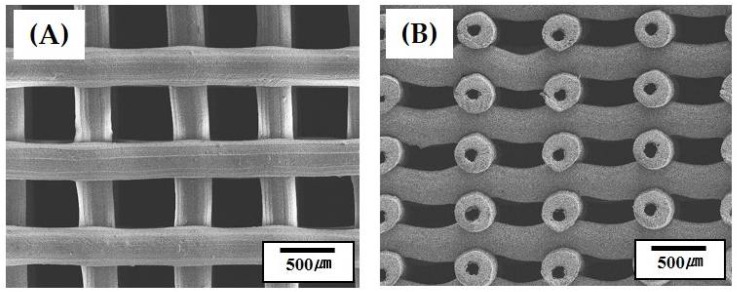
Representative SEM images of the porous structures of the porous CaP scaffold comprised of hollow filaments, which was produced using the distance of 0.75 mm between extruded filaments: (**A**) top view; and (**B**) side view.

The porous CaP scaffold showed a periodic arrangement of hollow CaP filaments without severe distortion or collapse, as shown in Figure 10A,B. It should be noted that the hollow filament showed a uniform cross-sectional profile, which would be attributed to the use of the green filaments with high green strength. A high compressive strength of ~4.8 MPa was achieved at a high porosity of ~82 vol % (cf. Table 5). This finding suggests that the overall porosity and mechanical properties of porous CaP scaffolds can be readily tuned by adjusting the distance between hollow CaP filaments.

### 3.9. In Vitro Biocompatibility of Porous Scaffold

**Figure 11:**
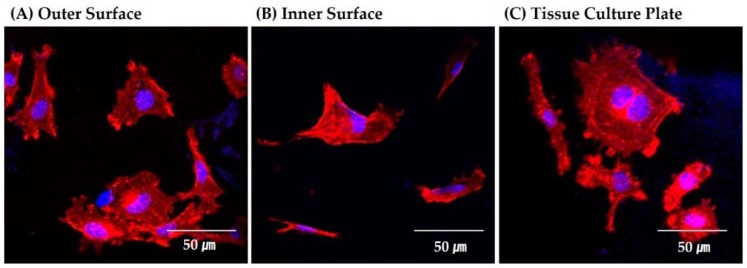
Representative CLSM images of the MC3T3 cells attached to: (**A**) the outer surface of the hollow filaments; (**B**) inner surface of the hollow filaments; and (**C**) the tissue culture plate used as the positive control.

To evaluate the potential of the porous CaP scaffold comprised of hollow filaments as the bone scaffold, its biocompatibility was assessed by in vitro cell tests using MC3T3-E1 cells. Figure 11A–C displays representative CLSM images of the MC3T3 cells attached to the outer and inner surfaces of the hollow filaments, and the tissue culture plate was used as the positive control. Basically, both outer and inner surfaces showed cellular responses similar to that of the tissue culture plate used as the positive control. That is, several cells adhered to and spread actively with the surfaces, where the red and blue colors represent the actin and nucleus, respectively. In addition, actin stress fibers, which is one of the main components of the cytoskeleton and plays a critical role in the control of many aspects of cellular activities, were vigorously organized. It should be noted that some cells migrated into the hollow filaments to some extent. This finding that the CB core used as the fugitive material for the creation of the channel would not deteriorate the excellent osteoblast activity of the CaP phase.

**Figure 12:**
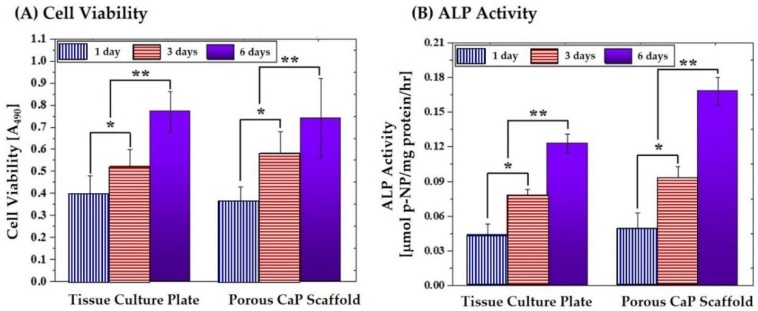
(**A**) Cell viability; and (**B**) ALP activity of the MC3T3-E1 cells that were cultured for various times on the porous CaP scaffolds comprised of hollow filaments and the tissue culture plate used as the positive control. (* *p* < 0.05 and ** *p* < 0.01)

The proliferation and differentiation of the MC3T3 cells were examined by MTS assay and ALP activity, as shown in Figure 12A,B, respectively. For comparison purposes, tissue culture plate was tested as the positive control. The porous CaP scaffold showed the level of cell viability and ALP activity comparable to those of the tissue culture plate after one day of cell culture. In addition, the level of cell viability and ALP activity increased continuously with an increase in a period of time, as shown in Figure 12A,B. This finding suggests that the porous CaP scaffold comprised of hollow CaP filaments can have good in vitro biocompatibility, indicating its great potential as the bone scaffold.

### 3.10. Potential as Framework for Porous Ceramic/Polymer Composite Scaffolds

**Figure 13:**
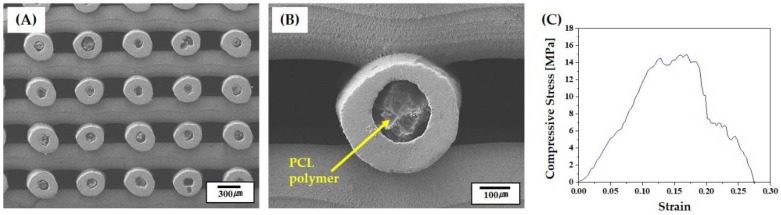
Representative SEM images of the PCL-infiltrated CaP scaffold at (**A**) low and (**B**) high magnifications; and (**C**) representative stress versus strain response during the compressive strength test.

Porous CaP scaffolds with hollow filaments can be used as the framework for the production of porous CaP/polymer composite scaffolds. More specifically, the channels in hollow filaments can be selectively infiltrated with biodegradable polymers, while preserving the three-dimensionally interconnected pores, thus enabling the designing and production of a new kind of ceramic/polymer composite scaffolds. To demonstrate this, the channels were filled with caprolactone oligomer, followed by thermal curing at 85 °C. Figure 13A,B shows representative SEM images of the PCL-infiltrated BCP scaffold. The channels in the hollow filaments were filled with the PCL polymer, while 3D pore networks were well preserved. This unique structure could effectively mitigate the brittle fracture of porous BCP scaffold, as shown in Figure 13C. That is, the porous BCP scaffold showed a rapid drop in stress after peak load due to its brittle fracture behavior (cf. Figure 8A). On the other hand, the PCL-infiltrated scaffold showed that the stress decreased gradually after peak load. In addition, it should be noted that drugs can be loaded into the PCL polymer, thus allowing for the production of porous BCP scaffold with the sustained release of drugs through thin BCP shells.

## 4. Conclusions

Porous CaP scaffolds comprised of hollow CaP filaments were successfully produced by the newly developed CoEx-3DP technique. A feedrod was specifically designed for coextrusion, which was comprised of the CB core and CaP shell with tailored extrusion behaviors, thus enabling extruded filaments to have a controlled core/shell structure. In addition, the rapid consolidation of extruded filaments through solvent extraction in acetone allowed for the 3D deposition of filaments with good shape tolerance. Produced CaP scaffolds showed a periodic arrangement of straight hollow filaments at an orientation of 0°/90°, as well as three-dimensionally interconnected pore networks (~360 × 230 μm^2^ in sizes). Owing to this unique porous structure, the porous scaffold could have a very high porosity of ~73 vol %. The fracture behavior and mechanical properties of the porous scaffolds were strongly affected by the alignment of hollow filaments. The porous CaP scaffold showed much higher compressive strength (~12.3 ± 2.2 MPa) when compressed parallel to the direction of hollow filaments, since the CaP shells could effectively withstand applied compressive load. The porous CaP scaffold showed good in vitro biocompatibility, where several cells spread actively with the outer and inner surfaces of hollow CaP filaments, and the level of cell viability and ALP activity increased continuously with an increase in a period of time. These findings suggest that our CoEx-3DP technique can produce a variety of porous ceramic scaffolds comprised of hollow filaments, which would have high porosity and high mechanical properties, thus finding very useful applications as bone scaffolds.

## Figures and Tables

**Table 1:** Compositions of the CaP and CB pastes used for the preparation of the CaP and CB pastes.

Components	CaP Paste	CB Paste
Powder Content	13.9 g (30 vol % in CaP Paste)	2.52 g (10 vol % in CB Paste)
Aqueous Solution	10 g (5 wt % MC solution)	10 g (5 wt % MC solution)
Dispersant (KD 6)	0.20 g (1.5 wt % in relation to CaP powder)	0.07 g (2.5 wt % in relation to CB powder)

**Table 2:** Dimensions of the green scaffold and porous CaP scaffold after sintering at 1250 °C for 2 h.

Scaffolds	Green Scaffold	Porous CaP Scaffold
Dimensions [mm]	~9.48 × 9.37 × 9.55	~6.52 × 6.43 × 6.58

**Table 3:** Dimensions of the 3D pores and diameters of channels formed in the designed, green, and sintered scaffolds.

Scaffolds	Designed Scaffold	Green Scaffold	Sintered Scaffold
Dimensions of 3D Pores (μm)	~500 × 440	~457 × 326	~360 × 230
Diameter of Channels (μm)	~500	~344	~187

**Table 4:** Compressive strengths of the porous CaP scaffolds comprised of hollow filaments, when compressed parallel and normal to the direction of hollow firmaments.

Loading Direction	Parallel	Normal
Compressive Strength (MPa)	12.3 ± 2.2	7.1 ± 1.9

**Table 5:** Compressive strengths of the porous CaP scaffolds with different porosities. The scaffolds were compressed normal to the direction of hollow firmaments.

**Overall Porosity (vol %)**	82 ± 2.2	73 ± 1.5
**Compressive Strength (Mpa)**	4.8 ± 1.1	7.1 ± 1.9
